# Cardiac fibroblast proliferation rates and collagen expression mature early and are unaltered with advancing age

**DOI:** 10.1172/jci.insight.140628

**Published:** 2020-12-17

**Authors:** Rimao Wu, Feiyang Ma, Anela Tosevska, Colin Farrell, Matteo Pellegrini, Arjun Deb

**Affiliations:** 1 Division of Cardiology, Department of Medicine, David Geffen School of Medicine,; 2 UCLA Cardiovascular Theme, David Geffen School of Medicine,; 3 Department of Molecular, Cell and Developmental Biology, College of Life Sciences,; 4 Eli & Edythe Broad Center of Regenerative Medicine and Stem Cell Research,; 5 Molecular Biology Institute,; 6 California NanoSystems Institute, and; 7 Department of Human Genetics, David Geffen School of Medicine, University of California, Los Angeles, California, USA.

**Keywords:** Aging, Cardiology, Cardiovascular disease, Extracellular matrix, Fibrosis

## Abstract

Cardiac fibrosis is a pathophysiologic hallmark of the aging heart, but little is known about how fibroblast proliferation and transcriptional programs change throughout the life span of the organism. Using EdU pulse labeling, we demonstrated that more than 50% of cardiac fibroblasts were actively proliferating in the first day of postnatal life. However, by 4 weeks, only 10% of cardiac fibroblasts were proliferating. By early adulthood, the fraction of proliferating cardiac fibroblasts further decreased to approximately 2%, where it remained throughout the rest of the organism’s life. We observed that maximal changes in cardiac fibroblast transcriptional programs and, in particular, collagen and ECM gene expression both in the heart and cardiac fibroblast were maximal in the newly born and juvenile animal and decreased with organismal aging. Examination of DNA methylation changes both in the heart and in cardiac fibroblasts did not demonstrate significant changes in differentially methylated regions between young and old mice. Our observations demonstrate that cardiac fibroblasts attain a stable proliferation rate and transcriptional program early in the life span of the organism and suggest that phenotypic changes in the aging heart are not directly attributable to changes in proliferation rate or altered collagen expression in cardiac fibroblasts.

## Introduction

The mammalian cardiac muscle cell is highly proliferative within the first few days of birth (1) but subsequently undergoes cell cycle arrest (2), and the myocyte undergoes minimal turnover for the rest of the life of the organism (3). As the organism transitions from immediate postnatal life to early adulthood and then to old age, age-related changes occur both in the cellular and extracellular components of the heart (4). Cardiac fibroblasts reside in an interstitial position between cardiomyocytes and are the principal cells that secrete extracellular matrix (ECM) (5). They exist in a quiescent state (6) in the uninjured heart; however, it is not clear whether fibroblasts exhibit an age-dependent increase in proliferation rate or increase in ECM gene expression that potentially contributes to age-related cardiac fibrosis. In fact, little is known about how cardiac fibroblast proliferation rates and transcriptional programs change from the immediate postnatal period through early adulthood and advanced age.

In this study, we investigate changes in cardiac fibroblast proliferation and transcription throughout the life span of the mouse, starting from day 1 of postnatal life through the early juvenile period (4 weeks), adulthood (14 and 24 weeks), and advanced age (1 and 1.5 years), to determine whether such age-dependent dynamic changes in the fibroblast could underlie the phenotype of increased fibrosis in the aging heart. We determined cardiac fibroblast proliferation rates in hearts of mice at different ages by measuring markers of proliferation or by pulse labeling of animals with 5-ethynyl-2′-deoxyuridine (EdU). Using these corroborative methodologies, we observed that the highest rates of cardiac fibroblast proliferation were in the immediate postnatal period, with subsequent decline in the rates of proliferation across early juvenile and early adulthood periods. By 14 weeks of age, the proliferative rates of cardiac fibroblasts had attained a low steady state of 2%–3% and despite advancing organismal aging, no further changes in cardiac fibroblast proliferation were observed. Concordant with age-related changes in cardiac fibroblast proliferation rate, we observed that there was no significant difference between the absolute numbers of cardiac fibroblasts between mice at 14 weeks of age and 1.5 years of age. We observed that the composition of the nonmyocyte cell population in the neonatal heart differs significantly from that of the juvenile or adult heart. In the neonatal heart, cardiac fibroblasts constituted most of the nonmyocyte cells and by early adulthood, with progressive decrease in fibroblast proliferation, endothelial cells formed most of the nonmyocyte cell fraction, which continued with organismal aging. Taken together, these observations demonstrate that cardiac fibroblasts attain a stable proliferation rate early in adulthood and do not exhibit an age-dependent increase in proliferation.

Collagens form the major component of the cardiac ECM. Because the aging heart is known to be more fibrotic, we investigated whether changes in fibroblast transcriptional programs and, in particular, collagen expression increases with age. Cardiac fibroblasts and hearts, from which cardiac fibroblasts were isolated, were harvested at different ages for gene expression analysis by RNA-Seq. Expression analysis of all collagens demonstrated that the highest collagen gene expression occurred in the immediate postnatal period, with a progressive decrease in collagen expression in juveniles, adults, and mice of advancing age. As epigenetic changes such as DNA methylation have recently been shown to be strong determinants of biological aging, we examined the degree of DNA methylation in cardiac fibroblasts isolated from early adulthood (14 weeks) and advanced age (1.5 years). However, we did not observe any significant differences in methylation. Our data suggest that cardiac fibroblasts do not exhibit an age-dependent increase in proliferation, collagen expression, or DNA methylation. Collectively, these results show that age-related cardiac phenotypes such as increased fibrosis cannot be directly attributed to altered fibroblast proliferation or increased ECM gene expression.

## Results

### EdU labeling of fibroblasts in hearts of neonatal, juvenile, adult, and old mice.

**Figure 1:**
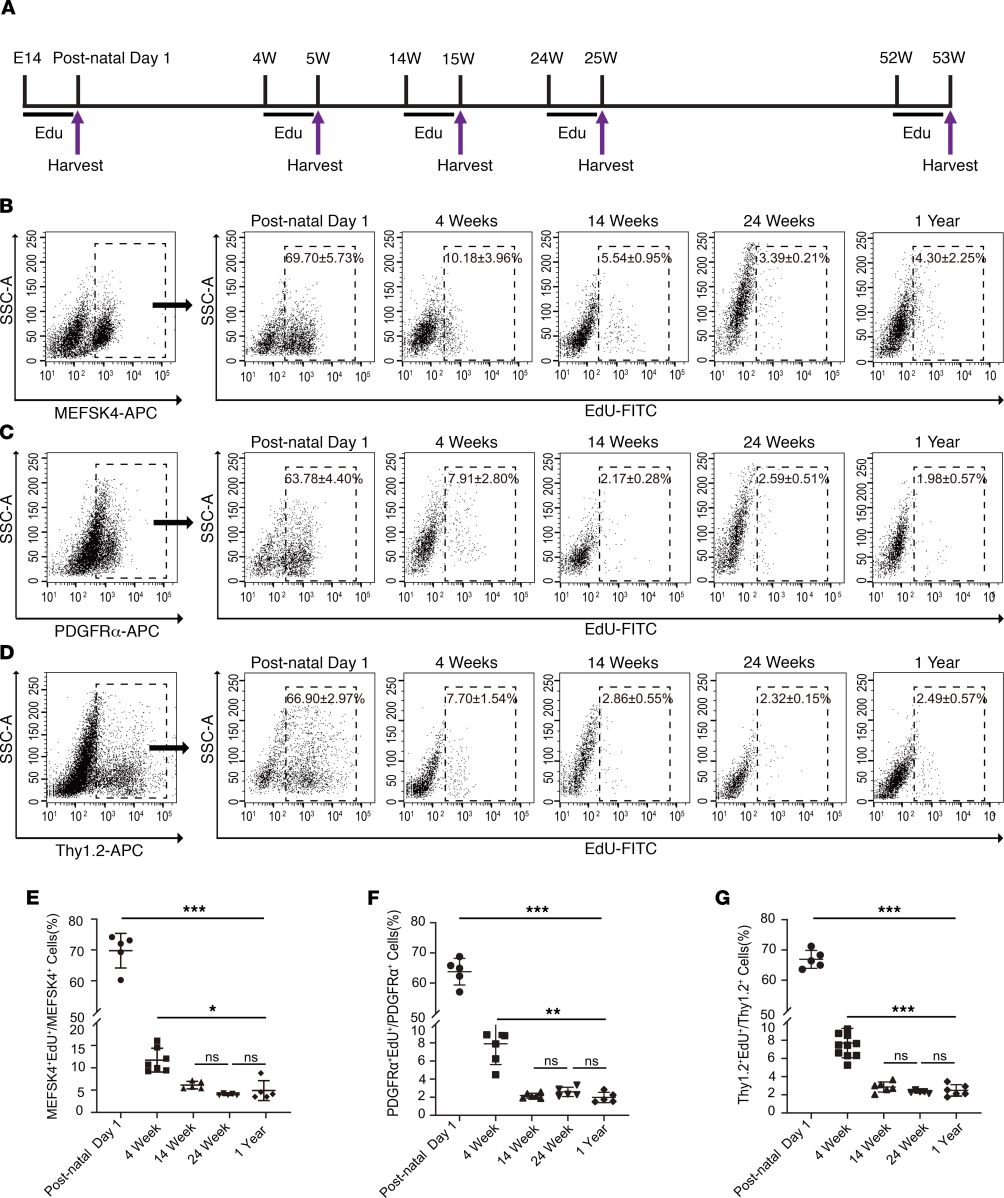
Cardiac fibroblast proliferation with advancing age assessed by 5-ethynyl-2′-deoxyuridine incorporation. (**A**) Experimental outline denoting timing of 5-ethynyl-2′-deoxyuridine (EdU) administration and subsequent harvest 7 days later. (**B–D**). The nonmyocyte cell fraction was first flow sorted according to expression of (**B**) MEFSK4, (**C**) PDGFRα, (**D**) Thy 1.2, and EdU uptake determined in each isolated fraction at each time point (postnatal day 1, 4 weeks, 14 weeks, 24 weeks, and 1 year). Quantitation of (**E**) MEFSK4, (**F**) PDGFRα, and (**G**) Thy1.2 cardiac fibroblasts that take up EdU at different ages. Postnatal day 1 value was compared with each value at 4 weeks, 14 weeks, 24 weeks, and 1 year, respectively. The 4-week value was similarly compared with each value at 14 weeks, 24 weeks, and 1 year. ***P < 0.0001, **P < 0.01, *P < 0.05, ns = *P* > 0.05. Data are represented as the mean ± SD. Data analysis was performed by 1-way ANOVA with multiple-comparisons correction.

To determine proliferation rates in cardiac fibroblast at various ages in postnatal life, we administered the thymidine analog EdU daily for 7 days to pregnant animals (7 days before delivery) or animals at 4 weeks, 14 weeks, 24 weeks, and 1 year of age (Figure 1A). At the completion of 7 days of daily EdU administration, the animals were euthanized and the hearts harvested and subjected to enzymatic digestion to remove myocytes and isolate the nonmyocyte fraction (7). We next subjected the nonmyocyte fraction to flow cytometry to determine the fraction of cardiac fibroblasts that retained EdU. Owing to the lack of a universal marker for cardiac fibroblasts, a panoply of cell surface antigens (PDGFRα, MEFSK4, and CD90.2 [Thy 1.2]) that are known to be abundantly expressed in cardiac fibroblasts were used for identifying cardiac fibroblasts (8, 9). Flow cytometry demonstrated that, on postnatal day 1, 69.70% ± 5.73% of MEFSK4 (Figure 1B), 63.78% ± 4.40% of PDGFRα (Figure 1C), and 66.90% ± 2.97% of Thy1.2 (Figure 1D) cardiac fibroblasts retained EdU (mean ± SD, *n* = 5 hearts). However, by 4 weeks of age, the number of cells that had taken up EdU during the prior 7 days of administration had dropped to 10.18% ± 3.96% for MEFSK4 cardiac fibroblasts (mean ± SD, *n* = 7 hearts), 7.91% ± 2.80% for PDGFRα (mean ± SD, *n* = 6 hearts), and 7.70% ± 1.54% for Thy1.2 cardiac fibroblasts, respectively (mean ± SD, *n* = 11) (Figure 1, B–D). At 14 weeks (mean ± SD, *n* = 5 [MEFSK4], *n* = 6 [PDGFRα, Thy1.2]) and 24 weeks (mean ± SD, *n* = 5), we observed a further decrease with approximately 2.1%–5.5% of cardiac fibroblasts being positive for EdU uptake (mean ± SD, *n* = 5). At 1 year of age, the fraction of cardiac fibroblasts that had taken up EdU was 4.30% ± 2.26% for MEFSK4, 1.98% ± 0.57% for PDGFRα, and 2.49% ± 0.57% for Thy1.2 (mean ± SD, *n* = 5), and there were no significant differences between the fraction of EdU-positive cardiac fibroblasts between 14 weeks, 6 months, and 1 year (Figure 1, E–G). Taken together, these observations suggest the highest rates of cardiac fibroblast proliferation are observed in the immediate postnatal period and that the proliferation rate rapidly declines by the early juvenile period (4 weeks) and significantly declines further to a stable and low level of cardiac fibroblast proliferation by early adulthood (14 weeks), remaining similar with advancing organismal age.

### Determination of cardiac fibroblast proliferation by assessment of proliferation markers.

**Figure 2:**
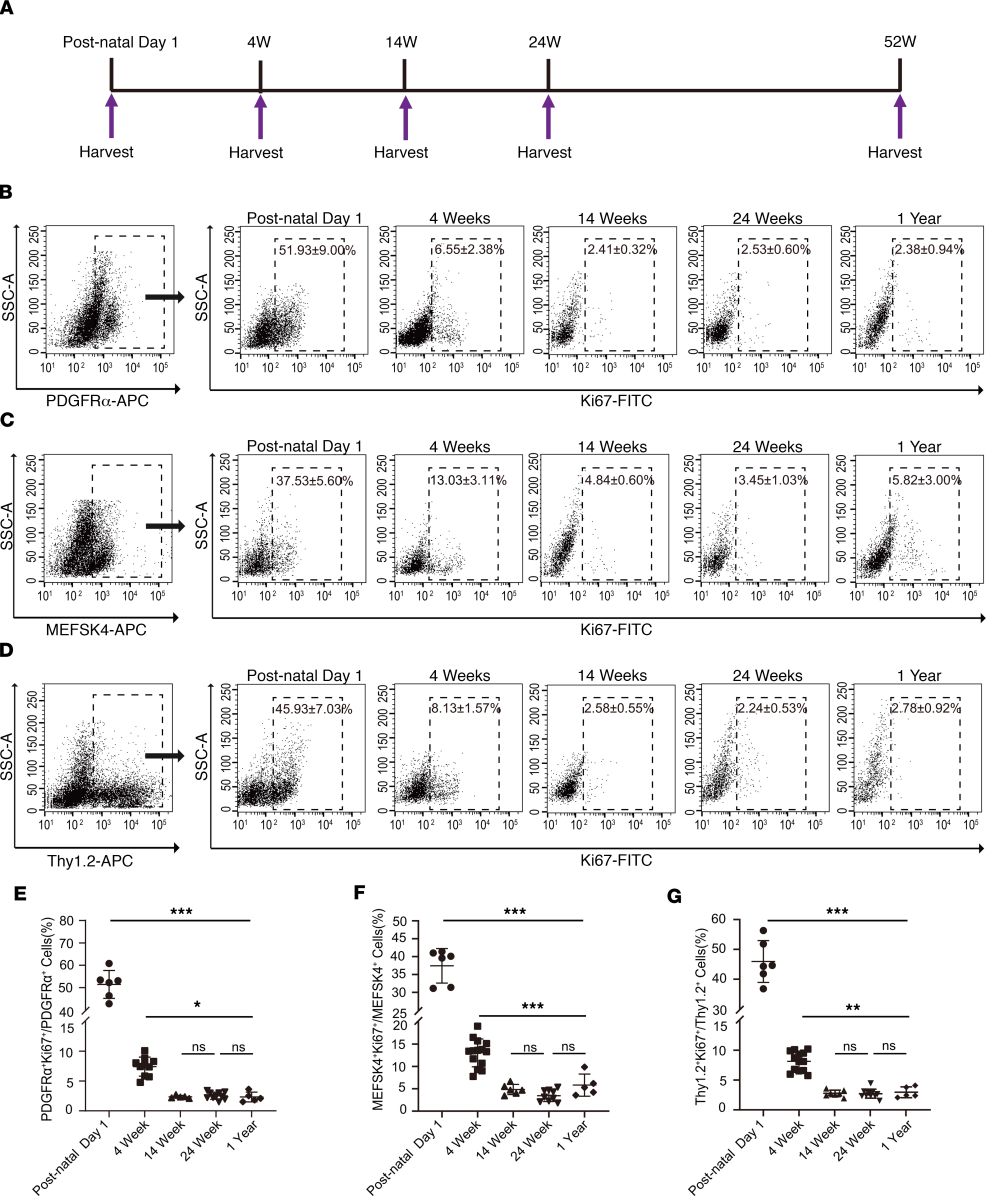
Cardiac fibroblast proliferation with advancing age assessed by Ki67 coexpression. (**A**) Schematic depicting timing of harvest of heart for cardiac fibroblast isolation. (**B–D**) Cardiac fibroblasts were sorted from the nonmyocyte cell population by flow sorting for (**B**) PDGFRα, (**C**) MEFSK4, and (**D**) Thy 1.2, and Ki67 expression in each of those fractions determined by flow cytometry at postnatal day 1, 4 weeks, 14 weeks, 24 weeks, and 1 year. Quantitation of (**E**) PDGFRα, (**F**) MEFSK4, and (**G**) Thy 1.2 cardiac fibroblasts that coexpressed Ki67 at different ages. Postnatal day 1 value was compared with each value at 4 weeks, 14 weeks, 24 weeks, and 1 year, respectively. The 4-week value was similarly compared with each value at 14 weeks, 24 weeks, and 1 year. ***P < 0.0001, **P < 0.01, *P < 0.05, ns = *P* > 0.05. Data are represented as the mean ± SD. Data analysis was performed by 1-way ANOVA with multiple-comparisons correction.

To corroborate our results with EdU uptake by cardiac fibroblasts, we determined marker expression of cardiac fibroblast proliferation. We adopted a similar experimental design and again harvested hearts of animals at postnatal day 1, 4 weeks, 14 weeks, 6 months, and 1 year of age; isolated cardiac fibroblasts; and used the same markers to determine the fraction of cardiac fibroblasts expressing Ki67, a marker of proliferation (Figure 2A). We observed that the highest rates of proliferation occurred at postnatal day 1, with 51.93% ± 9% of PDGFRα, 37.53% ± 5.6% of MEFSK4, and 45.93% ± 7.03% of Thy1.2 cardiac fibroblasts coexpressing Ki67 (mean ± SD, *n* = 6 hearts) (Figure 2, B–D). Overall, these numbers agree with the fraction of cardiac fibroblasts that had taken up EdU at postnatal day 1 and suggest that more than one-half of cardiac fibroblasts are actively proliferating at postnatal day 1. At 4 weeks of age, 6.55% ± 2.38% of PDGFRα (mean ± SD, *n* = 12 hearts), 13.03% ± 3.11% of MEFSK4 (mean ± SD, *n* = 14 hearts), and 8.13% ± 1.57% of Thy1.2 (mean ± SD, *n* = 12 hearts) cardiac fibroblasts coexpressed Ki67 (Figure 2, B–D). These observations mirror the significant decreases in the fraction of proliferating cardiac fibroblasts from postnatal day 1 to 4 weeks seen with EdU administration. At 14 weeks of age, the fraction of proliferating cardiac fibroblasts expressing Ki67 had significantly decreased further to between 2.4% and 4.8% (mean ± SD, *n* = 5 [PDGFRα], *n* = 7 [MEFSK4, Thy1.2]); however, beyond 14 weeks, the rate of cardiac fibroblast proliferation again remained similar, with no significant differences in the fraction of proliferating cardiac fibroblasts between 14 weeks, 6 months, and 1 year of age (Figure 2, E–G).

### Fraction of cardiac fibroblasts comprising the nonmyocyte population from postnatal day 1 to 1 year of age.

**Figure 3:**
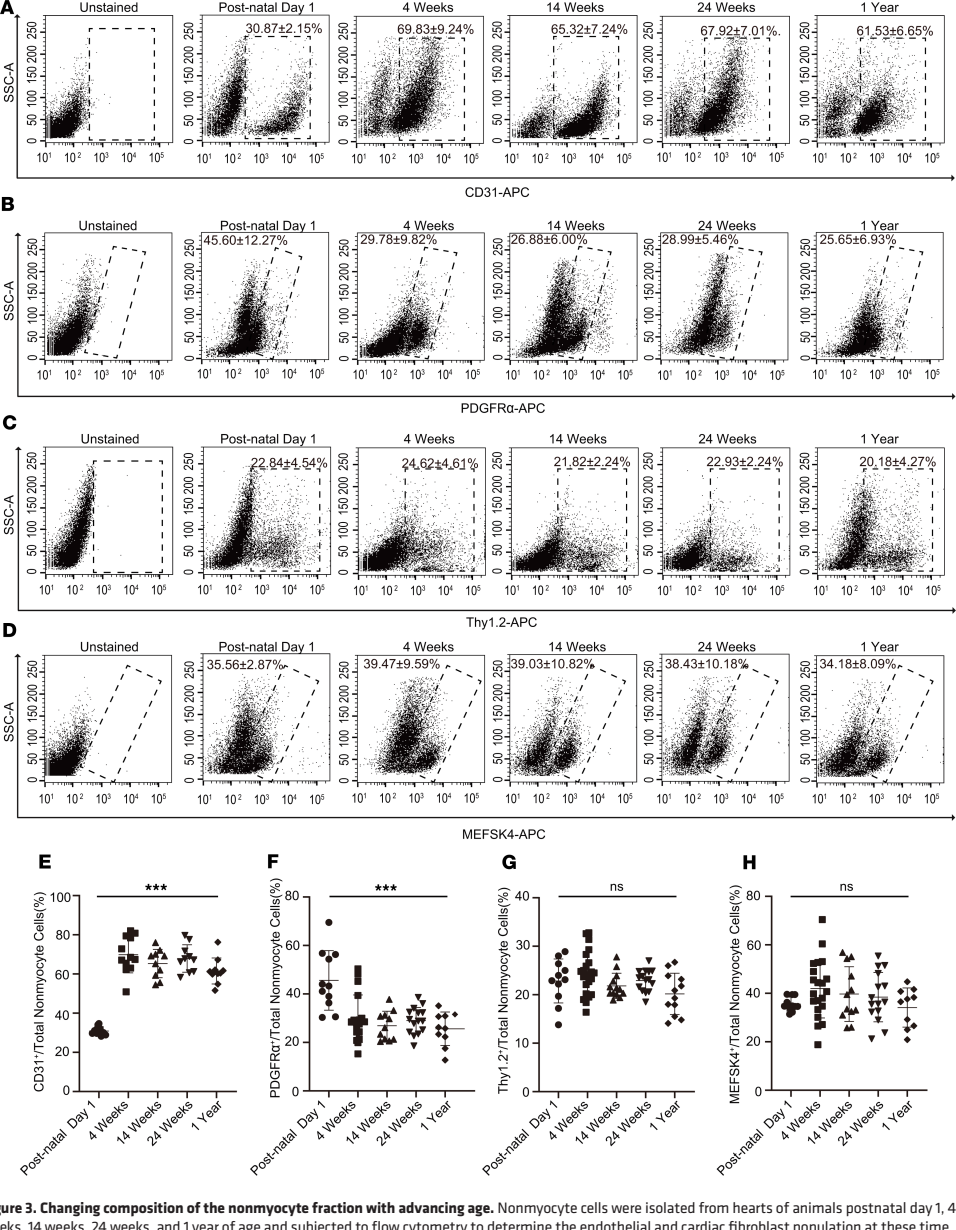
Changing composition of the nonmyocyte fraction with advancing age. Nonmyocyte cells were isolated from hearts of animals postnatal day 1, 4 weeks, 14 weeks, 24 weeks, and 1 year of age and subjected to flow cytometry to determine the endothelial and cardiac fibroblast population at these time points. (**A–D**) Flow cytometry demonstrating fraction of nonmyocytes staining for (**A**) CD31 (endothelial), (**B**) PDGFRα, (**C**) Thy1.2, and (**D**) MEFSK4, and (**E–H**). Quantitation of (**E**) CD31, (**F**) PDGFRα, (**G**) Thy1.2, and (**H**) MEFSK4 expressing cells as a fraction of the entire nonmyocyte population isolated from the hearts at those time points. Postnatal day 1 value was compared with each value at 4 weeks, 14 weeks, 24 weeks, and 1 year, respectively. ***P < 0.0001, ns = *P* > 0.05. Data are represented as the mean ± SD. Data analysis was performed by 1-way ANOVA with multiple-comparisons correction.

We next determined the fraction of cardiac fibroblasts constituting the cardiac nonmyocyte cell population. Emerging evidence suggests that cardiac fibroblasts in the adult animal comprise approximately 20% of the nonmyocyte fraction, with endothelial cells comprising most of the nonmyocytes in the adult heart (9). We performed flow cytometry for endothelial marker (CD31) and observed that CD31-expressing cells made up only 30.87% ± 2.15% of nonmyocyte cells at postnatal day 1 (mean ± SD, *n* = 10 hearts); however, the fraction of endothelial cells by 4 weeks had increased to approximately 69.83% ± 9.24% of the nonmyocyte population (mean ± SD, *n* = 10 hearts) and remained close to 65% at 14 weeks, 24 weeks, and 1 year (Figure 3, A and E). Our data are consistent with recent reports that endothelial cells represent the predominant nonmyocyte population in the adult heart (9). By contrast, the population of PDGFRα-expressing fibroblasts as a fraction of the nonmyocyte population was approximately 45.60% ± 12.27% at postnatal day 1 (mean ± SD, *n* = 10 hearts) but had decreased to 29.78% ± 9.82% at 4 weeks (mean ± SD, *n* = 23 hearts) and remained between 25% and 30% of the nonmyocyte population in adults and mice of advancing age (Figure 3, B and F). The fraction of Thy1.2 and MEFSK4 cells constituting the nonmyocyte fraction did not change significantly from neonatal to adult life and beyond, suggesting that cardiac fibroblasts identified by PDGFRα expression undergo the most dynamic changes in numbers, from neonatal life to adulthood (Figure 3, C, D, G, and H). Our results show that the composition of the nonmyocyte cell population of the neonatal heart is significantly different from that observed in the adult heart; however, by 4 weeks of age, the cellular composition of cardiac nonmyocytes is very similar to that in the adult heart, and the fraction of cardiac fibroblasts constituting the nonmyocyte fraction does not significantly change with advancing age (Figure 3, E–H) (mean ± SD, *n* = 10 hearts).

**Figure 4:**
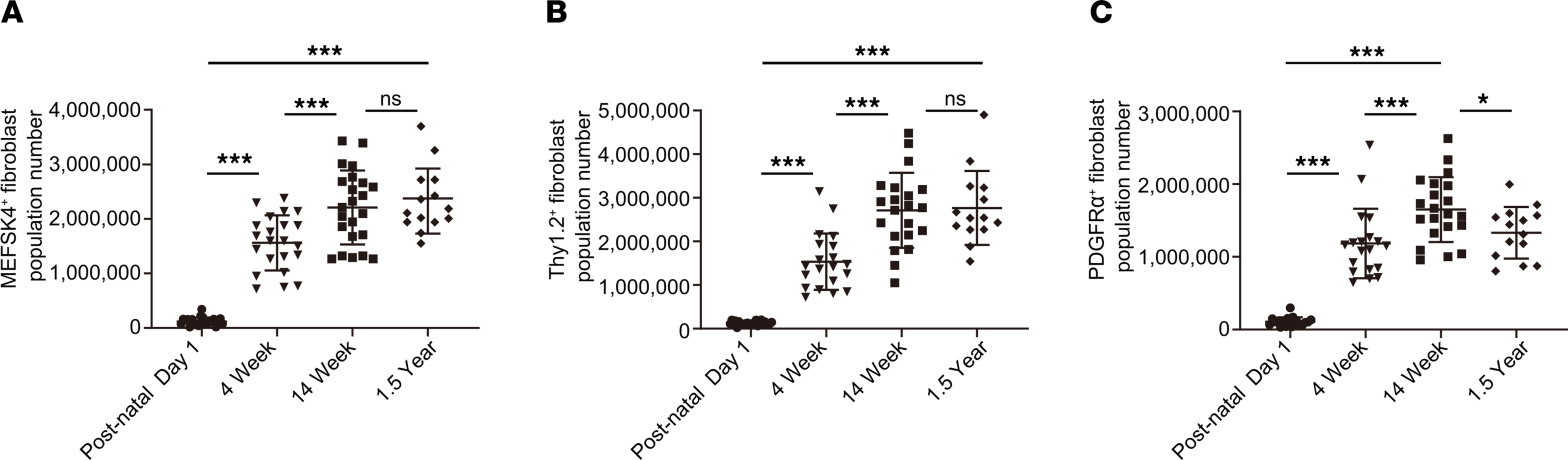
Absolute cardiac fibroblast numbers in hearts of mice determined by beads-based flow cytometry. Quantitation of absolute numbers of (**A**) MEFSK4, (**B**) Thy1.2, and (**C**) PDGFRα cardiac fibroblasts in animal hearts of postnatal day 1, 4 weeks, 14 weeks, and 1.5 years of age. ***P < 0.0001, *P < 0.05, ns = *P* > 0.05. Data are represented as the mean ± SD. Data analysis was performed by 1-way ANOVA with multiple-comparisons correction.

Next, we examined the absolute numbers of cardiac fibroblasts in hearts of mice from the neonatal period to old age to determine whether the changes in the absolute numbers of cardiac fibroblasts were concordant with age-dependent changes in cardiac fibroblast proliferation rates. To determine the absolute numbers of cardiac fibroblasts, we harvested whole heart tissue from animals postnatal day 1 to 1.5 years of age, isolated the cardiac fibroblasts by MEFSK4, Thy1.2, and PDGFRα expression, and normalized the number of cardiac fibroblasts to the total number of cells in unit weight of heart tissue as previously described (10). We observed that the number of MEFSK4, Thy1.2, and PDGFRα cardiac fibroblasts at 4 (mean ± SD, *n* = 20 hearts) and 14 weeks (mean ± SD, *n* = 21 hearts) of age was significantly greater than that in animal hearts of postnatal day 1 (mean ± SD, *n* = 21 [MEFSK4], *n* = 20 [PDGFRα], *n* = 26 [Thy1.2]) and 4 weeks of age, respectively (Figure 4, A–C). However, there was no significant difference in absolute cardiac fibroblast numbers between animals 1.5 years (mean ± SD, *n* = 14 hearts) and 14 weeks of age with the absolute numbers of PDGFRα cardiac fibroblasts, demonstrating a slight but significant decrease at 1.5 years compared with 14 weeks (Figure 4, A–C). Therefore, these data are concordant with the age-dependent changes observed in cardiac fibroblast numbers. Cardiac fibroblasts are highly proliferative in the immediate postnatal period and remain proliferative at 4 weeks of age, albeit at a lower proliferative rate. Therefore, the absolute numbers of cardiac fibroblasts are higher at 4 weeks compared with postnatal day 1 and at 14 weeks compared with 4 weeks. However, the proliferation rate declines thereafter and remains stable for the rest of the life of the organism, and absolute cardiac fibroblast numbers do not change significantly thereafter with age.

We also investigated whether there were sex-specific differences in absolute cardiac fibroblast proliferation rates. Examination of the absolute numbers of MEFSK4-expressing cardiac fibroblasts did not show any differences between male and female mice at postnatal day 1, 4 weeks, 14 weeks, or 1.5 years of age (Supplemental Figure 1A; supplemental material available online with this article; https://doi.org/10.1172/jci.insight.140628DS1). Similarly, there were no differences in the proliferation rates of Thy1.2-expressing cardiac fibroblasts (as measured by Edu uptake) between male and female animals at different ages (Supplemental Figure 1B).

### Gene expression changes in cardiac fibroblasts and the heart from postnatal to advanced age.

**Figure 5:**
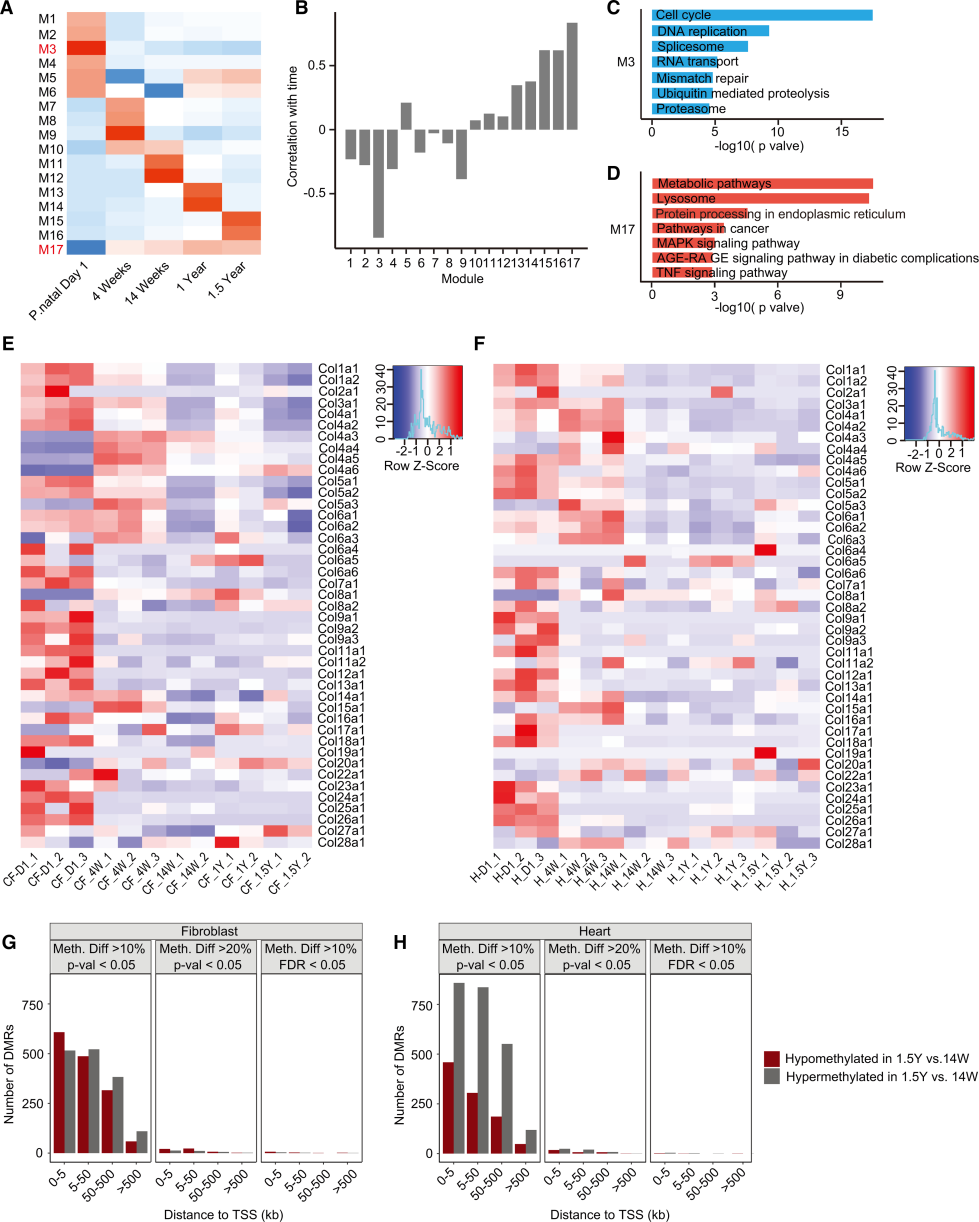
Transcriptional programs and DNA methylation of cardiac fibroblasts and heart with advancing age. Hearts were harvested from animals at postnatal day 1, 4 weeks, 14 weeks, 1 year, and 1.5 years of age, and the heart tissue as well as PDGFRα fibroblasts isolated from those hearts were subjected to gene expression analysis by RNA-Seq. (**A**) Weighted gene coexpression network analysis of genes (WGCNA) was performed to identify modules of genes that correlated with time and a heatmap demonstrates mean expression of these modules of genes with age (**B**). Graphical representation demonstrating correlation of each module with time (**C** and **D**). Gene ontology analysis demonstrating principal pathways/biological processes that are (**C**) downregulated or (**D**) upregulated with age. (**E** and **F**) Heatmap demonstrating changes in expression of all collagen encoding genes with age in (**E**) cardiac fibroblasts (PDGFRα^+^) and (**F**) whole heart tissue. (**G** and **H**) Bar plot showing the number of differentially methylated regions (DMRs) between (**G**) cardiac fibroblasts (PDGFRα^+^) and (**H**) animal hearts at 1.5 and 14 weeks of age, binned by the absolute distance to the nearest transcription start site (TSS); DMRs (hyper- and hypomethylated) are shown both in fibroblasts and whole heart tissue. Left panel shows regions with at least 10% difference in methylation between 1.5-year and 14-week samples and a *P* value of less than 0.05 (Mann-Whitney *U* test); middle panel shows regions with at least 20% difference in methylation between 1.5-year and 14-week samples and a *P* value of less than 0.05 (Mann-Whitney *U* test); right panel shows number of sites that are retained after adjusting for FDR (<0.1) with a minimum of 10% difference in methylation between 1.5-year and 14-week samples (*n* = 3 hearts or 3 sets of cardiac fibroblasts for each time point).

**Figure 6:**
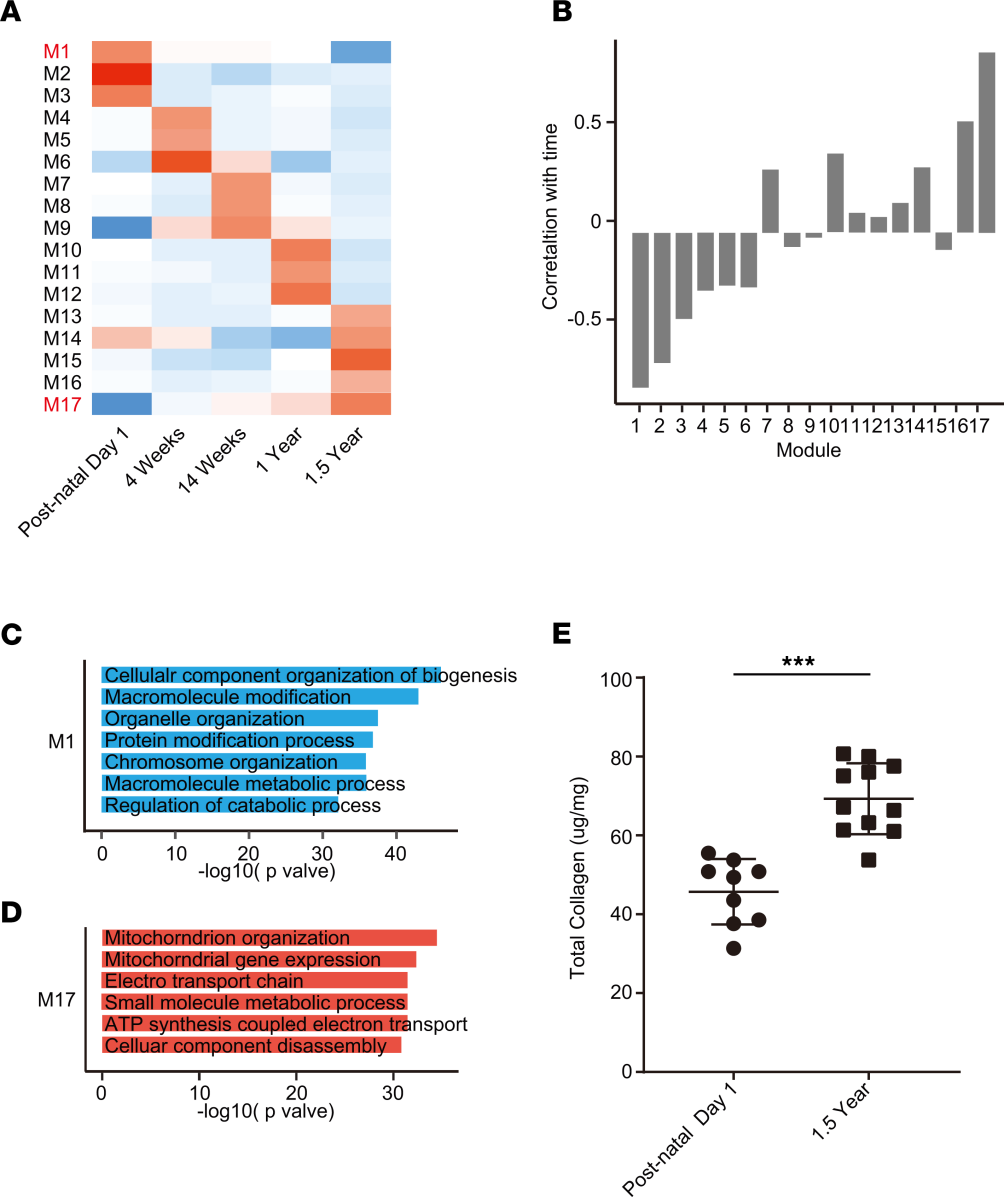
Transcriptional programs and collagen content of young and old hearts. (**A**) Hearts were harvested from animals at postnatal day 1, 4 weeks, 14 weeks, 1 year, and 1.5 years of age, and the heart tissue subjected to gene expression analysis by RNA-Seq. (**A**) WGCNA was performed to identify modules of genes that correlated with time, and a heatmap demonstrates mean expression of these modules of genes in whole heart tissue with age. (**B**) Graphical representation demonstrating correlation of each module with time in the heart. (**C** and **D**) Gene ontology analysis demonstrating principal pathways/biological processes that are (**C**) downregulated or (**D**) upregulated with age in module M1 or M17, respectively. (**E**) Total collagen content of animal hearts postnatal day 1 and 1.5 years of age, expressed as unit weight of heart tissue. ***P < 0.001. Data are represented as the mean ± SD. Data analysis was performed by 2-tailed Student’s *t* test.

As the heart ages, there is accumulation of ECM or fibrosis that is considered to be characteristic phenotype of cardiac aging. Because cardiac fibroblasts do not exhibit an age-dependent increase in proliferation, we next investigated how the transcriptional program of cardiac fibroblasts changes from immediate postnatal life to advanced age. For this purpose, we harvested hearts from animals at postnatal day 1, 4 weeks, 14 weeks, 1 year, and 1.5 years of age. Cardiac fibroblasts were isolated by PDGFRα expression, and both cardiac fibroblasts and the hearts from which they were isolated were subjected to gene expression analysis by RNA-Seq. First, we analyzed differentially expressed genes (DEGs) among PDGFRα-expressing cardiac fibroblasts from all age groups (i.e., from postnatal day 1 to 1.5 years) and performed weighted gene correlation network analysis (WGCNA) to find modules of genes highly correlated with advancing age (Figure 5A). Of the modules described, we observed that the genes in modules 3 and 17 (M3 and M17) exhibited the strongest correlation with advancing age (Figure 5B). M3 represented a cluster of genes that was progressively downregulated with advancing age, and gene ontology analysis demonstrated genes associated with cell cycle and DNA replication to be predominantly downregulated, consistent with our observations of decreased cell proliferation of cardiac fibroblasts with age (Figure 5C and Table 1). A smaller cluster of genes (M17) demonstrated age-dependent increase in expression in cardiac fibroblasts and represented genes regulating metabolic pathways, MAPK, and inflammation (Figure 5D and Table 2). A similar analysis of genes that correlate with advancing age in the whole heart demonstrated a module of genes (M1) to be progressively downregulated with age (Figure 6, A and B), and this contained a diverse set of genes regulating cellular and organelle organization, protein modification, and associated metabolic processes (Figure 6C and Table 3). Clusters of genes that show progressive upregulation with advancing age (M17) in the whole heart included genes that regulated mitochondrial function, including electron transport chain, ATP synthesis, and mitochondrial organization (Figure 6D and Table 4).

Because the aging heart is known to be more fibrotic, we determined collagen expression in the heart as well in PDGFRα fibroblasts isolated from animals at postnatal day 1 to 1.5 years of age. We observed that most collagen gene expression, including the genes for the principal collagens Col I (Col1a1, Col1a2, and Col1a3) and Col III (Col3a1), significantly decreased from the neonatal period to 4 weeks and beyond (Figure 5E). Only genes encoding for Col IV, a collagen present in the basement membrane of blood vessels showed increased expression from postnatal day 1 to 4 weeks of age, consistent with our observations of more endothelial cells at 4 weeks of age (Figure 5E). Analysis of collagen expression in the whole heart mirrored that of cardiac fibroblasts, with the principal collagen encoding genes for Col1 and Col III exhibiting the highest expression in postnatal day 1 and undergoing downregulation of expression with age (Figure 5F). We examined the total collagen content of hearts. Consistent with previous reports of increased collagen content in the aging heart (11), we determined the 1.5-year-old heart had 51% more collagen than the postnatal day 1 heart (69.3 ± 9.01 μg/mg in 1.5-year-old hearts [*n* = 11] vs. 45.7 ± 8.3 μg/mg in postnatal day 1 hearts [*n* = 9], mean ± SD) (Figure 6E). Therefore, these data suggest that although collagen expression in the heart and the cardiac fibroblasts significantly decreases with age, the heart accumulates collagen protein with age. Nascent collagen undergoes cross-linking with age, and cross-linked collagen is resistant to breakdown by collagenases or other matrix metalloproteinases (12). We examined age-related changes in gene expression in both cardiac fibroblasts and the heart to determine if there were any patterns in expression of MMPs, tissue inhibitors of metalloproteinases (TIMPs), or genes regulating cross-linking (lysyl oxidase). We observed that several MMPs such as MMP3 and MMP 19 as well as TIMP1 and TIMP2 showed significant increased expression in older mice compared with younger animals (Supplemental Figure 2, A and B). However, no significant pattern was obvious with downregulation of both members of the MMP and TIMP family in both cardiac fibroblasts and hearts of older mice compared with younger animals (Supplemental Figure 2, A and B). As the TGF-β pathway is known to be a critical regulator of fibrosis, we examined gene expression in the TGF-β pathway and observed decreased age-dependent decrease in Smad1 and TGFBR 1 expression in both the heart and cardiac fibroblasts; however, no obvious pattern was evident with increased expression of other members of the TGF, SMAD, and BMP family (Supplemental Figure 2, C and D). We also looked at lysyl oxidase (LOX) expression, which regulated cross-linking of ECM, and observed significant downregulation of gene expression with age both in the heart and in cardiac fibroblasts with age (Supplemental Figure 2, E and F). Collectively, these data suggest that the accumulation of collagen in the aging heart is not directly linked to increased collagen expression or to increased numbers of cardiac fibroblasts but likely represent mechanisms of decreased turnover owing to cross-linking or decreased proteolysis.

Epigenetic changes such as changes in DNA methylation have been recently considered strong indicators of aging in a variety of tissues (13). Our data show that cardiac fibroblast proliferation rates as well as transcription programs become stable by 14 weeks and do not undergo any further changes. We next determined the DNA methylation status of PDGFRα-expressing cardiac fibroblasts and whole hearts harvested from animals at 14 weeks and 1.5 years of age, respectively. We examined the differentially methylated regions (DMRs) with respect to hypo/hypermethylation and binned them according to distance from transcription start sites. More than 2500 regions in the genome of cardiac fibroblasts harvested at 14 weeks and 1.5 years of age exhibited at least 10% difference in methylation. However, the number of regions that exhibited at least 20% differences in the degree of methylation dropped to less than 100 and applying a stringent criterion of FDR less than 0.1, we observed very few regions exhibiting any differences (Figure 5G). Examination of DMR in the whole heart tissue of animals 14 weeks and 1.5 years of age also mirrored observations seen in cardiac fibroblasts, and there were minimal differences in the DMRs when stringent statistical criteria were employed (Figure 5H). These observations demonstrate that, even with advancing age, the methylation status of the genome of both cardiac fibroblasts and the heart does not appreciably change beyond 14 weeks of age.

## Discussion

Our data show the dynamics of fibroblast proliferation and transcriptional programs from early postnatal life to advanced age. The aging heart is known to have a greater amount of fibrotic tissue, but fibroblast proliferation rates are the greatest in the neonatal period and rapidly decline thereafter. These results are consistent with previously reported observations demonstrating that fibroblast proliferation rates rapidly decline within the first few weeks of postnatal life (14). Fibroblast numbers calculated as the fraction of nonmyocyte cells or even absolute cardiac fibroblast numbers did not significantly differ in young and old hearts, and thus changes in fibroblast proliferation likely do not directly underlie increased fibrosis seen with aging. We used antigens abundantly expressed in cardiac fibroblasts to identify broad populations of cardiac fibroblasts in the heart. Although age-dependent proliferation rates were similar in the fibroblast populations examined, it is possible that subsets of cardiac fibroblasts within these populations or others not examined by us could differ in age-dependent biological properties. Consistent with age-dependent decline or stabilization of low proliferation rates, expression of key collagen genes is the highest in the neonatal period and stabilizes to a low rate by early adulthood, suggesting that fibroblasts maintain a quiescent fibrotic program throughout life that is stable from early adulthood. The mechanisms of age-dependent fibrosis are poorly understood (15). However, the increased amount of ECM in the aging heart cannot be directly attributed to increased fibroblast proliferation rates or increased collagen expression, resulting from the decreased breakdown of collagen, as collagen gets more cross-linked and more resistant to proteolysis. In this regard, we observed that LOX expression decreased from the neonatal to the adult mouse and could reflect decreased gene expression as the matrix gets more cross-linked. Whether the increased accumulation of collagen in the aging heart exerts a negative feedback effect on cardiac fibroblasts to decrease collagen expression remains unclear. Rather than being a cell that contributes to increased collagen deposition with age, our data suggest that the cardiac fibroblast, by decreasing collagen expression and lowering proliferation rates from early adulthood to advanced age, potentially plays a protective role in preventing excessive fibrosis of the heart with organismal aging.

## Methods

### Isolation of nonmyocyte population from the heart.

C57BL/6 mice from postnatal day 1 to 1.5 years of age and hearts were harvested. The ventricles were used for isolation of nonmyocyte cells following removal of the atria and the heart valves. The hearts were rinsed in ice-cold HBSS chopped into 1 mm square pieces, suspended in 0.1 μg/mL liberase TH (Roche, 5401151001) in Tyrode’s buffer (136 mM NaCl, 5.4 mM KCl, 0.33 mM NaH2PO4, 1 mM MgCl2, 10 mM HEPES, and 0.18% glucose), and subsequently placed in a shaking incubator at 37˚C for 30 minutes at 80 rpm. Digested hearts were filtered with a 40 μm cell strainer (Fisher, 22363547), centrifuged at 200*g* for 5 minutes, and nonmyocyte cell resuspended with 10 mL 1% BSA.

### Flow cytometry for fibroblast and endothelial antigens, EdU uptake, and Ki67 expression.

C57BL/6 animals were used for all experiments. For determination of EdU incorporation, either pregnant animals (7 days before birth of pups) or animals at 4 weeks, 14 weeks, 24 weeks, and 1 year of age were administered EdU daily (Carbosynth, NE0870) at 50 mg/kg for 7 consecutive days. Following completion of EdU administration, animals were euthanized and hearts harvested for isolation of nonmyocyte cells. For identification of cardiac fibroblasts, 2 × 10^6^ nonmyocytes suspended in 100 μL 1% BSA in PBS were incubated for 30 minutes with fluorophore-conjugated antibodies, targeting antigens abundantly present in cardiac fibroblasts MEFSK4-APC (1:20, Miltenyi Biotec 130-102-302), PDGFRα-APC (1:20, eBioscience 17-1401-81), and Thy1.2-APC (1:200 eBioscience 17-0902-82). For identification of endothelial cells, nonmyocyte cells were incubated with CD31-APC (1:50, eBioscience 17-0311-82). For identification of EdU uptake, the Click-iT Edu Alexa Fluor 488 flow cytometry assay kit (Invitrogen, C10425) was used according to the manufacturer’s instructions, and flow cytometry performed to identify MEFSK4, PDGFRα, and Thy1.2 nonmyocyte cell fractions that also were positive for EdU uptake. For identification of cardiac fibroblasts that also coexpressed Ki67 (1:200, eBioscience 11-5698-82), the nonmyocyte fraction was first stained with MEFSK4 and then followed by fixation with 4% paraformaldehyde, permeabilization (eBioscience, 00-8333-56), and staining for Ki67. For PDGFRα and Thy1.2, the cells were fixed and permeabilized first, stained with Ki67, then stained for surface markers. BD LSRII flow cytometer was used for all flow cytometry experiments. Data were analyzed using FlowJo software. Supplemental Table 1 shows a summary of reagents.

### Quantitation of absolute numbers of cardiac fibroblasts.

Absolute numbers of cardiac fibroblasts were determined using a bead-based quantification assay. The nonmyocyte cell population was isolated as previously described. All cells were then resuspended in 1% BSA in PBS, and then the cellular suspension was spiked with Calibration Particles (Sphero 556298) at a concentration of 1000 beads/milligram of absolute tissue weight (measured before processing). Cardiac fibroblasts were identified by staining with fluorescence-conjugated cardiac fibroblast antibodies against cell-specific antigen markers. Invitrogen Attune NxT Flow Cytometer was used to count the cell number data analysis by FlowJo. Calculation of absolute cell numbers was performed as follows using the 2 equations below using the beads/volume of tissue for normalization as previously described (10): number of gated cells/numbers of gated beads × total beads added to the sample/mass of sample (mg) = cells/mg.tissue and absolute cell number = cells/mg.tissue × tissue weight.

### Total collagen assay.

Assessment of collagen content in postnatal day 1 and 1.5-year-old hearts was performed using Sircol Soluble Collagen Assay Kit (Biocolor, S1000) and Sircol Insoluble Collagen Assay Kit (Biocolor, S2000) to determine total collagen content. Tissues were weighed and homogenized in 0.1 mg/mL pepsin/0.5 M acetic acid and subsequently incubated overnight at 4°C in the same buffer. Lysates were centrifuged at 12,000*g* for 10 minutes. Supernatants were used to measure soluble collagen, and precipitates were used to detect insoluble collagen. For soluble collagen, the supernatants were transferred to new tubes, mixed with 1 mL Sircol Dye Reagent, and incubated for 30 minutes with gentle shaking. Precipitates were washed with 750 μL ice-cold Acid-Salt Wash Reagent. Washed precipitates were dissolved in 500 μL Alkali Reagent, 200 μL each sample was added to a 96-well plate, and absorbance measured at 550 nm using Synergy H1 microplate reader (BioTeK). Soluble collagen standards were added to create a standard curve for quantitative readouts. For determination of insoluble collagen, tissue precipitates were incubated with Fragmentation Reagent at 65°C for 2 hours with vigorous mixing by vortexing every 30 minutes to convert insoluble collagen to soluble collagen. Treated samples were centrifuged at 12,000*g* for 10 minutes, supernatants were transferred into new tubes, and collagen content estimated as for soluble collagen.

### Determination of gene expression changes in cardiac fibroblasts and hearts isolated from animals from postnatal day 1 to 1.5 years.

Animals at postnatal day 1, 4 weeks, 14 weeks, 1 year, and 1.5 years of age were used for determination of gene expression analysis. PDGFRα cardiac fibroblasts were isolated by flow cytometry from hearts of animals at the above respective ages to determine changes in gene expression in cardiac fibroblasts. RNA was isolated from the heart tissue or cardiac fibroblasts using RNeasy Mini Kit (QIAGEN) and used to generate RNA-Seq libraries followed by sequencing using Illumina 4000 platform (single-end, 65 bp). The reads were mapped with STAR 2.5.3a (16) to the mouse genome (mm10). The counts for each gene were obtained by using –quantMode GeneCounts commands in STAR, and the other parameters during alignment were set to default. Differential expression analyses were carried out using DESeq2 (17). Normalized counts were obtained using the DESeq2 rlog function with default parameters. Three animals/time point and 3 different sets of PDGFRα fibroblasts isolated from different hearts at each time point were used for differential expression analysis.

Module analysis of correlation networks was performed using WGCNA (18). The rlog transformed counts from DESeq2 were used in the WGCNA analysis. A signed gene correlation network was constructed using the soft thresholding power of 10. The module eigengene, which represents a linear combination of genes that capture a large fraction of variance in each module, was used to calculate correlation with each time. The modules with the most positive and negative correlation were retained, and genes in these modules were used for enrichment analysis by g:Profiler (19). The accession number for the RNA sequencing data described in this study is GEO GSE 161079.

### DNA methylation analysis.

Hearts of animals 14 weeks and 1.5 years of age were used for determination of differences in DNA methylation of either PDGFRα cardiac fibroblasts isolated from those hearts or whole heart tissue. DNA was first extracted from whole hearts or cardiac fibroblasts (QIAGEN All prep DNA/RNA mini kit, catalog 80204) and then subjected to using Illumina NovaSeq SP platform (pair-end, 150bp). Reads were aligned against the mouse genome mm10 using BiSulfite Bolt (https://bsbolt.readthedocs.io/en/latest/), followed by methylation calling. Methylation matrix was constructed using only CpG sites with at least 10× coverage across at least 80% of the samples. DMRs were defined using metilene v02-8 (20). DMRs were defined as regions with at least 3 CpG sites and at least 10% of methylation difference between comparisons at *P* value of less than 0.05 in the Mann-Whitney *U* test. The Benjamini-Hochberg procedure was used to adjust the FDR. HOMER v4.11 (21) was used for DMR annotation to the human genome (hg38). The accession number for the DNA methylation sequencing data described in this study is GEO GSE 161079.

### Statistics.

All statistical and graphical analyses were performed using GraphPad Prism 8. Data represent the mean ± SD. Significant differences between groups were calculated using 2-tailed Student’s *t* test. Analysis of differences between more than 2 groups was performed using a 1-way ANOVA with multiple-comparisons correction in GraphPad. A *P* value of less than 0.05 was considered significant.

### Study approval.

All experiments were approved by the Animal Research Committee at the University of California, Los Angeles.

## Author contributions

RW performed all experiments. FM analyzed the RNA-Seq data. AT and CF analyzed the DNA methylation data. MP oversaw the RNA-Seq and methylation data analysis. AD conceptualized the project, supervised the data collection and analysis, and wrote the manuscript.

## Supplementary Material

Supplemental data

## Figures and Tables

**Table 4:**
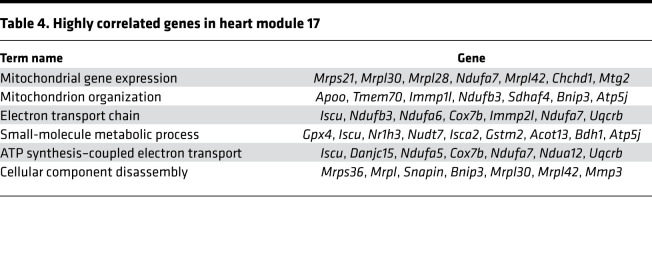
Highly correlated genes in heart module 17

**Table 3:**
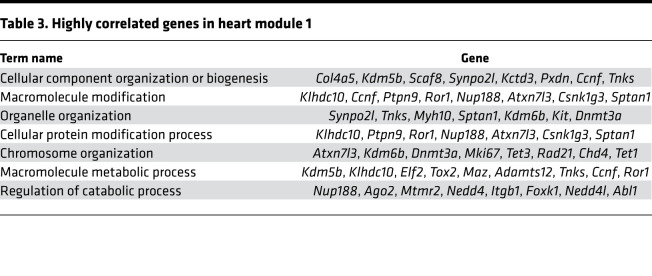
Highly correlated genes in heart module 1

**Table 1:**
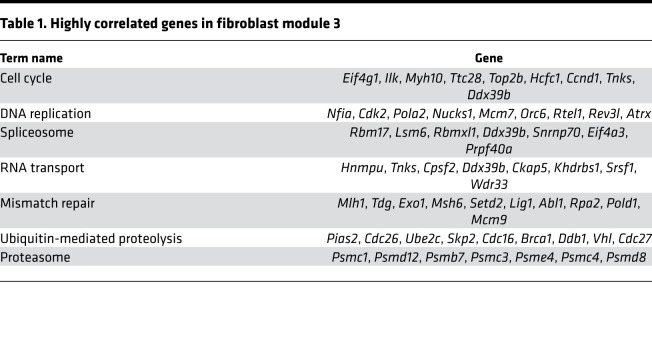
Highly correlated genes in fibroblast module 3

**Table 2:**
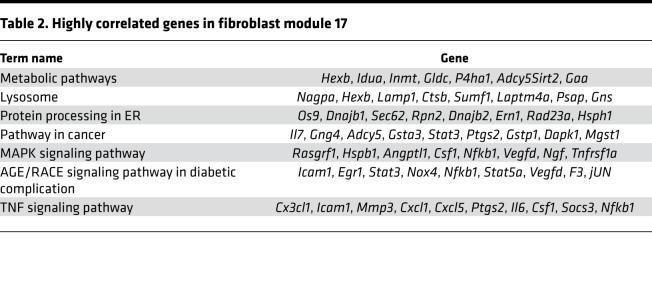
Highly correlated genes in fibroblast module 17

## Version 1 11/12/2020

In-Press Preview

## Version 2 12/17/2020

Electronic publication
