# Supplementary material for: Cardiac fibroblast proliferation rates and collagen expression mature early and are unaltered with advancing age
Source: JCI Insight. 2020 Dec 17;5(24):e140628. doi: 10.1172/jci.insight.140628 (PMC7819745; doi:10.1172/jci.insight.140628)
Supplement: Supplemental data [file jciinsight-5-140628-s133.pdf]

A

Fig.S1

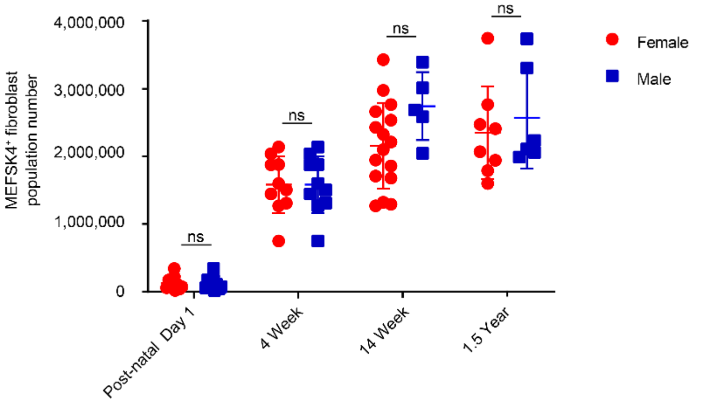

B

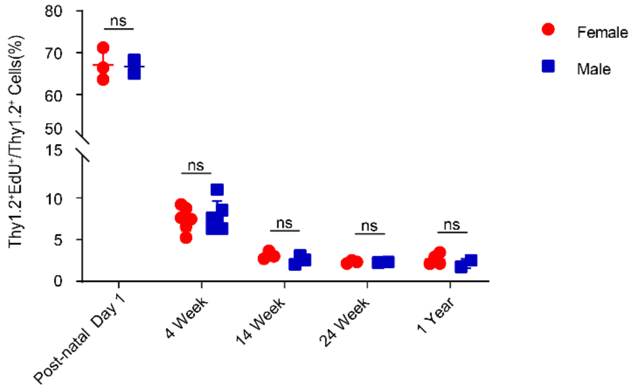

Supplementary Figure 1. Absolute cardiac fibroblast numbers and fibroblast proliferation rates in male and female mice of different ages. (A) Hearts were harvested from male and female mice of different ages and absolute cardiac fibroblast numbers measured between male and female hearts at each age (Neonatal n=11 Females and10 Males; 4 weeks n=10 Females and 10 Males; 14 weeks n=16 Female and 5 Males; 1.5 years n=8 Females and 6 Males; mean ± S.D.; ns= p>0.05). **(B)** Both male and female mice of different ages were injected with EdU (daily for 1 week) followed by harvesting of hearts and determination of numbers of Thy1.2EdU+ cells as a fraction of Thy1.2 cells in male and female hearts (Neonatal n=3 Females and 2 Males; 4 weeks n= 6 Females and 6 Males; 14 weeks n=3 Females and 3 Males; 24 weeks n=3 Females and 2 Males; 1.5 year n= 4 Females and 2 Males; mean ± S.D.; ns= p>0.05) Data analysis was performed by 1-way ANOVA with multiple-comparisons correction.

Fig.S2

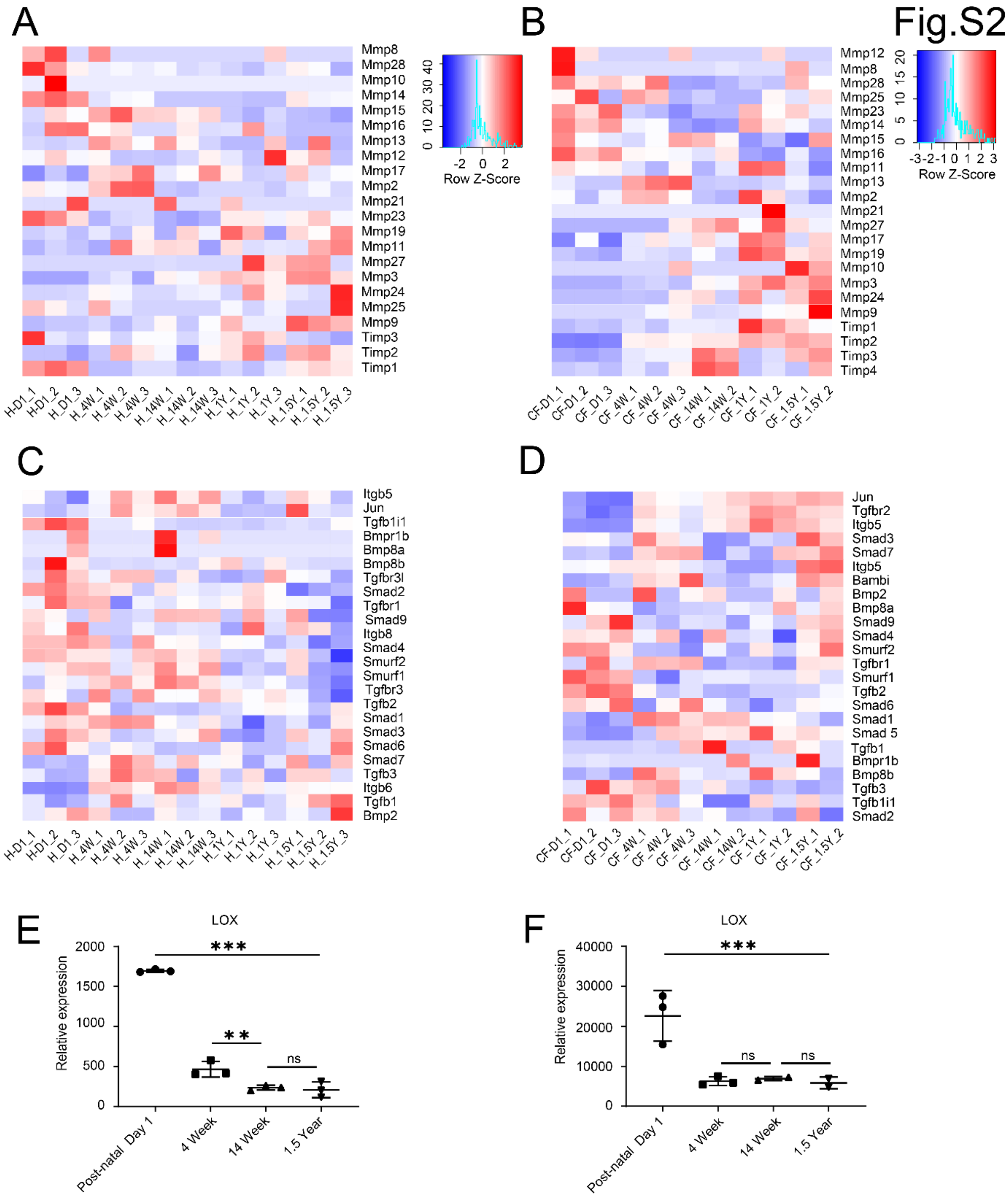

Supplementary Figure 2. **Temporal changes in heat expression of MMP, TIMP and LOX in hearts and cardiac fibroblasts of mice of different ages**. **(A,B)** Heat map demonstrating expression of members of the MMP family in **(A)** hearts and **(B)** cardiac fibroblasts isolated from hearts of mice at different ages (H: Heart CF: Cardiac fibroblasts). **(C,D)** Heat map demonstrating expression of members of the TGF-β signaling pathway in **(C)** hearts and **(D)** cardiac fibroblasts isolated from hearts of mice of different ages. (H: Heart CF: Cardiac fibroblasts). **(E,F)** Gene expression of lysyl oxidase (LOX) in hearts and cardiac fibroblasts isolated from hearts of mice of different ages (mean ± S.D.; *** p<0.0001, **p<0.01, ns= p>0.05). Data analysis was performed by 1-way ANOVA with multiple-comparisons correction.

Supplementary Table 1

REAGENT or RESOURCE	SOURCE	IDENTIFIER
Antibodies		
MEFSK4-APC	Miltenyi Biotec	130-102-302
PDGFR-a-APC antibody	eBioscience	17-1401-81
Thy1.2-APC antibody	eBioscience	17-0902-82
CD31-APC antibody	eBioscience	17-0311-82
Ki 67-488 antibody	eBioscience	11-5698-82
Critical Commercial Kit and Reagent		
Permeabilization Buffer	eBioscience	00-8333-56
Click-iT™ Edu Alexa Fluor 488 flow cytometry assay kit	Invitrogen	C10425
Edu	Carbosynth,	NE0870
Calibration Particles	BD Sphero	556298
Sircol Soluble Collagen Assay Kit	Biocolor	S1000
Sircol Insoluble Collagen Assay Kit	Biocolor	S2000
Qiagen All prep DNA/RNA mini kit	Qiagen	80204
